# Cursos en línea abiertos y masivos: oportunidad de aprendizaje sobre salud global en Latinoamérica

**DOI:** 10.7705/biomedica.6582

**Published:** 2023-06-30

**Authors:** Guido Bendezu-Quispe, Brenda Caira-Chuquineyra, Daniel Fernandez-Guzman, Reggie Casanova-Pérez, Andrés Guido Bendezú-Martínez

**Affiliations:** 1 Escuela de Medicina, Universidad César Vallejo, Trujillo, Perú Universidad César Vallejo Escuela de Medicina Universidad César Vallejo Trujillo Peru; 2 Facultad de Medicina, Universidad Nacional de San Agustín de Arequipa, Arequipa, Perú Universidad Nacional de San Agustín Facultad de Medicina Universidad Nacional de San Agustín Arequipa Peru; 3 Carrera de Medicina Humana, Universidad Científica del Sur, Lima, Perú Universidad Científica del Sur Carrera de Medicina Humana Universidad Científica del Sur Lima Peru; 4 University of Washington, Seattle, WA, USA University of Washington University of Washington Seattle WA USA; 5 Facultad de Medicina Humana, Universidad Nacional San Luis Gonzaga, Ica, Perú Universidad Nacional San Luis Gonzaga Facultad de Medicina Humana Universidad Nacional San Luis Gonzaga Ica Peru

**Keywords:** aprendizaje, autoaprendizaje como asunto, educación a distancia, salud global, educación continua, educación en salud, Learning, global health, self-directed learning as topic, education, distance, education, continuing, health education

## Abstract

**Introducción.:**

Los cursos en línea, masivos y abiertos, brindan la oportunidad de formar profesionales e investigadores en Latinoamérica sobre salud global.

**Objetivos.:**

Determinar la oferta global de los cursos en línea, masivos y abiertos, sobre salud global y conocer las características de su contenido.

**Materiales y métodos.:**

Se examinaron las plataformas especializadas en cursos en línea, masivos y abiertos, para recopilar aquellos sobre salud global. La búsqueda no tuvo restricción de tiempo y se realizó por última vez en noviembre de 2021. La estrategia de búsqueda solo incluyó el descriptor “global health”. Posteriormente, se obtuvieron las características del curso, su contenido y el dominio abordado de salud global. Estos datos fueron analizados descriptivamente, y se reportaron frecuencias absolutas y relativas.

**Resultados.:**

La estrategia de búsqueda identificó 4.724 cursos en línea, masivos y abiertos. De ellos, solo 92 estaban relacionados con salud global. La mayoría de estos cursos (n=44; 47,8 %) se ofrecieron mediante la plataforma Coursera. Más de la mitad de los cursos (n=50; 54,4 %) fueron realizados por instituciones de Estados Unidos y en idioma inglés (n=90; 97,8 %). La mayor parte de los cursos se centró en la “globalización de la salud y la asistencia sanitaria” (n=24; 26,1 %), seguido de los dominios “fortalecimiento de capacidades” (n=16; 17,4 %), “carga global de enfermedad” y “determinantes sociales y ambientales de la salud” (n=15; 16,3 %).

**Conclusiones.:**

Se encontró una importante oferta de cursos en línea, masivos y abiertos, sobre salud global. Estos cursos abordaron las competencias de la salud global que se requieren para los profesionales sanitarios.

## Abstract (en)

**Introduction.:**

Massive open online courses provide the opportunity to train health professionals and researchers from Latin America in global health.

**Objective.:**

To determine the global offer of massive open online courses in global health and the characteristics of their content.

**Material and methods.:**

We examined massive open online course platforms to compile the global health offerings. The search had no time restriction and was last conducted in November, 2021. The search strategy only included the descriptor “global health”. We obtained the characteristics of the courses, their content, and the global health domain covered. These data were analyzed using descriptive statistics, reporting absolute and relative frequencies.

**Results.:**

Our search strategy identified 4,724 massive open online courses. Of these, only 92 were related to global health. Most of these courses (n=44; 47.8%) were offered through Coursera. More than half (n=50; 54.4%) of the MOOCs were conducted by U.S.A. institutions and in English language (n=90; 97.8%). Most courses focused on “globalization of health and healthcare” (n=24; 26.1%), followed by the domains “capacity building” (n=16; 17.4%), “global burden of disease” and “social and environmental determinants of health” (n=15; 16.3%).

**Conclusions.:**

We found a high offer of massive open online courses on global health. These courses covered the global health competencies required for health professionals.

## Introducción

La salud global es un área de estudio, investigación y práctica, con pocos años de introducción, encargada de priorizar problemas, cuestiones y preocupaciones sanitarias que trascienden las fronteras nacionales y que se abordan mejor mediante acciones y soluciones cooperativas ^1^. Además, busca lograr la equidad en salud en todas las regiones del mundo por medio de un enfoque multidisciplinario, multisectorial y culturalmente sensible ^2^^,^^3^. La salud global aborda la reducción de disparidades y vela por la protección contra las amenazas para la salud mundial como la creciente carga de morbilidad, las barreras de acceso a los medicamentos, el cambio climático y los brotes de enfermedades emergentes y reemergentes ^4^^-^^6^.

Por ende, la enseñanza de esta área es de importancia para los estudiantes y médicos de todas las regiones, quienes enfrentarán las consecuencias de lascrisis globales y deberán plantear medidas preventivas y de solución para disminuir tales impactos ^7^^,^^8^. No obstante, en las últimas décadas, la educación sobre salud global solo ha crecido en los programas de especialización de países de Europa y Norteamérica ^9^, mientras que en países en desarrollo, como los de Latinoamérica, su enseñanza recién está en crecimiento ^7^.

La educación virtual ha sido una estrategia importante para el aprendizaje de conocimientos y habilidades en diferentes áreas, siendo los cursos en línea, masivos y abiertos una de las herramientas más útiles. Estos cursos ofrecen acceso a educación con estándares de alta calidad (respaldados por universidades, institutos o empresas) a un gran número de participantes de todo el mundo, con acceso a internet y a bajo costo o sin costo alguno ^10^. Los cursos pretenden acercar a los estudiantes a nuevas disciplinas, estimular nuevos intereses, generar ideas y crear una comunidad de aprendizaje ^10^, al ofrecer una amplia gama de temáticas: desde ciencias básicas hasta especializaciones en salud ^11^.

Desde su aparición, estos cursos se han extendido rápidamente en todo el mundo favorecidos por el mayor uso y acceso a internet ^12^. Hasta el 2020 se habían contabilizado i8o millones de participantes en 16.300 cursos, de los cuales el 7,7 % corresponde a salud y medicina ^13^. Los cursos han facilitado la enseñanza de múltiples áreas de la salud ^14^, a pesar de esto y la calidad demostrada en los estándares de educación virtual, su incorporación y uso es desigual, lenta y heterogénea en Latinoamérica ^15^^,^^16^, por las dificultades de acceso a la tecnología, las barreras del idioma y una baja contribución por parte de instituciones latinoamericanas, especialmente en ciencias de la salud ^17^.

Se ha descrito que la enseñanza de salud global tiene el propósito de alcanzar competencias en la carga global de la enfermedad, globalización de la salud y la asistencia sanitaria, determinantes sociales y ambientales de la salud, fortalecimiento de la capacidad, colaboración, asociación y comunicación; se enfoca en la ética, la práctica profesional, la equidad sanitaria y la justicia social, la gestión de programas, la conciencia sociocultural y política, y el análisis estratégico ^18^. Sin embargo, los planes de estudio de programas sobre salud global varían mucho en los tópicos del curso, la calidad del contenido y los costos ^8^, siendo impartidos, sobre todo, en países de altos ingresos ^9^^,^^19^.

En Latinoamérica, la oferta académica en salud global es limitada, siendo pocas las instituciones que ofrecen cursos o programas de pregrado o posgrado en esta área. Se destacan experiencias limitadas en Chile, México y Perú ^20^. Ante la necesidad de formación en salud global en países de la región, los cursos en línea representan una herramienta útil para formar profesionales e investigadores en el campo de la salud global. A pesar de ello, no se ha descrito en la literatura la oferta educativa de estos cursos sobre salud global.

Por tanto, el objetivo de este estudio fue evaluar la oferta global de los cursos en salud global y conocer las características de su contenido, así como las oportunidades, las barreras y las lecciones ofrecidas en las plataformas que existen hasta la fecha (noviembre de 2021) para la formación continua de profesionales. Se espera que el auge de los ellos pueda ser útil como herramienta para la formación continua y equitativa de los profesionales de todo el mundo en salud global.

## Materiales y métodos

Se llevó a cabo un estudio descriptivo para identificar la oferta de cursos en línea sobre salud global y caracterizar sus contenidos. Para ello, se hizo una búsqueda de los cursos en línea sobre salud global entre el 15 y el 21 de noviembre de 2021 en plataformas de aprendizaje virtual que los ofrecen.

Dado que no existen bases de datos que integren sistemáticamente los cursos en línea de diferentes plataformas, se realizó una búsqueda individual en los siguientes sitios: Coursera, edX, FutureLearn, Canvas Network, Miriadax, iversity, Blackboard Open Education, NovoEd, Udacity, Crypt4you, Alison, Stepik, FUN MOOC, openHPI, NPTEL, Independent, UPVX, OpenWHO, Udemy y XuentangX. Además, te, se hizo una búsqueda en dos sitios web que recopilan cursos de distintas plataformas cursos en línea: Class-Central y MOOC List. El empleo de las anteriores plataformas de cursos en línea para identificar aquellos sobre salud global se sustenta en su uso previo para el estudio de la oferta de cursos en línea en otros temas relacionados con salud ^11^^,^^14^^,^^17^^,^^21^.

Se diseñó una estrategia de búsqueda utilizando como guía los términos DeCS y MeSH para “Global Health”. En la primera etapa, se utilizaron estos términos de búsqueda en inglés, debido a que, hasta la fecha, el mayor número de cursos y plataformas de cursos en línea se encuentran en ese idioma ^22^. Posteriormente, se hizo una búsqueda complementaria con el término “salud global” para localizar los cursos ofrecidos en español en las plataformas en inglés y en aquellas que solo los ofrecen en español.

Los criterios empleados para la inclusión de los cursos en línea en este estudio fueron: un enfoque total o parcial sobre la salud global y contar con el respaldo de instituciones académicas o científicas, independientemente del idioma, año de publicación, o si el curso era o no gratuito.

Dos de los autores realizaron un tamizaje independiente de cada uno de los cursos encontrados como resultado de las búsquedas en las distintas plataformas revisadas, siguiendo los criterios de inclusión. En caso de discrepancias, un tercer autor resolvió la inclusión o no de los cursos para el estudio. Una vez identificados, se procedió a la identificación y eliminación de duplicados con base en la comparación de los títulos, los autores y la institución que los ofrece.

Luego de la eliminación de duplicados, se recopilaron las siguientes características de cada curso sobre salud global, registrando los datos en una hoja de cálculo del *software* Microsoft Excel: plataforma que ofrece los cursos en línea, institución y país de la institución, idioma disponible de audio y subtítulos/transcripción, duración y horas estimadas por semana, dominio de la salud global principalmente abordado (acorde con lo propuesto por el *Global Health Competency Subcommittee* del *Consortium of Universities for Global Health*) ^18^.

Se excluyeron los cursos sobre salud global que no tenían la información sobre las características recopiladas en este estudio. Finalmente, los datos registrados sobre cada curso fueron exportados al *software* Stata™, versión 16.0, para el análisis descriptivo y reportar las frecuencias absolutas y relativas.

## Resultados

De los 4.724 cursos encontrados con la estrategia de búsqueda aplicada en las distintas plataformas, 139 estaban relacionados con salud global. De estos últimos, se excluyeron 47 por ser duplicados. Con ello, se analizaron las características de 92 cursos en línea sobre salud global.

La mayoría de los cursos incluidos se encontraron en la plataforma Coursera (n=44; 47,8 %), seguido de FutureLearn (n=18; 19,6 %) y EdX (n=16; 17,4 %). Norteamérica, como región de origen, representó más de la mitad de los cursos encontrados (n=52; 56,5 %), seguido de Europa (n=37; 40.2 %). No se encontraron cursos en línea sobre salud global en Latinoamérica, ni en África; se identificaron únicamente dos de origen asiático y uno perteneciente a Australia (Oceanía).

A nivel de país, 50 (54,4 %) de los cursos en línea fueron realizados por instituciones de Estados Unidos; el Reino Unido (n=23; 25,0 %) y Francia (n=6; 6,5 %) fueron los otros dos países con mayor número de cursos sobre salud global. Respecto a las instituciones generadoras, Johns Hopkins University (n=17; 18,5 %) fue la que realizó el mayor número de cursos en línea sobre salud global, seguida de la Universidad de Coventry y la Universidad de Michigan, cada una con ocho cursos (8,7 %) (cuadro 1).

**Cuadro 1:** Características de los cursos en línea relacionados con salud global (n=92)

Características		Frecuencia	Porcentaje
Plataforma			
	Coursera	44	47,8
	FutureLearn	18	19,6
	EdX	16	17,4
	Alison	5	5,4
	FUN MOOC	3	3,3
	iversity	2	2,2
	Independent	2	2,2
	Udemy	2	2,2
Región			
	Norteamérica	52	56,5
	Europa	37	40,2
	Asia	2	2,2
	Australia	1	1,1
País			
	Estados Unidos	50	54,4
	Reino Unido	23	25,0
	Francia	6	6,5
	Australia	3	3,3
	Dinamarca	3	3,3
	Otros*	7	7,6
Institución			
	Johns Hopkins University	17	18,5
	Coventry University	8	8,7
	University of Michigan	8	8,7
	Imperial College, London	7	7,6
	Harvard University	5	5,4
	University of Geneva	3	3,3
	Otras*	44	47,9

* Menos de tres cursos en línea

En cuanto al idioma, 90 (97,8 %) de los cursos se ofrecieron con audio en inglés y sólo dos cursos (2,2 %) en francés. Respecto a la opción de transcripción o subtítulos de los cursos, se encontraron diez idiomas disponibles: 32 cursos (34,8 %) estaban subtitulados en español, portugués, francés y ruso; y otras opciones de subtítulos, menos frecuentes, fueron el árabe (n=12; 13,0 %), el italiano, el vietnamita y el alemán (n=11; 12,0 %) y el chino (n=3; 3,3 %) (cuadro 2).

**Cuadro 2:** Características de los contenidos de los cursos en línea relacionados con salud global (n=92)

Características		Frecuencia	Porcentaje
Idioma del curso (audio)			
	Inglés	90	97,8
	Francés	2	2,2
Idioma de los subtítulos			
	Inglés	61	66,3
	Francés	32	34,8
	Español	32	34,8
	Portugués	32	34,8
	Ruso	32	34,8
	Árabe	12	13,0
	Italiano, vietnamita o alemán	11	12,0
	Chino	3	3,3
Forma parte de un programa de especialización			
	No	56	60,9
	Sí	36	39,1
Duración*			
	Duración del curso (semanas)	5	4 - 7
	Tiempo estimado por semana (horas)	4,4	3 - 10
Acceso			
	Gratuito	73	79,4
	Pagado	19	20,7
Dominios en salud global			
	Globalización de la salud y la asistencia sanitaria	24	26,1
	Fortalecimiento de capacidades	16	17,4
	Carga global de enfermedad	15	16,3
	Determinantes sociales y ambientales de la salud	15	16,3
	Equidad sanitaria y justicia social	8	8,7
	Conciencia sociocultural y política	6	6,5
	Gestión de programas	4	4,4
	Práctica profesional	2	2,2
	Ética	1	1,1
	Análisis estratégico	1	1,1

* Mediana (RIC)

La mediana de la duración de los cursos fue de cinco semanas [rango intercuartílico (RIC): 4-7 semanas] mientras que la mediana del tiempo requerido por semana para completarlos fue de 4,4 horas (RIC: 3-10 horas).

De los cursos incluidos, 73 (79,4 %) eran de acceso libre y el dominio de salud más abordado fue “globalización de la salud y la asistencia sanitaria” (n=24; 26,1 %), seguido de “fortalecimiento de capacidades” (n=16; 17,4 %), “carga global de la enfermedad” y “determinantes sociales y ambientales de la salud” (n=15; 16,3 %). Los dominios de la salud global con menos cursos ofertados fueron ética y análisis estratégico (n=1; 1,1 % para ambos cursos).

## Discusión

El propósito de este estudio fue caracterizar la oferta de cursos en línea sobre salud global. Luego de la revisión en distintas plataformas que ofrecen cursos en línea, se encontraron cerca de 100 cursos con contenido sobre salud global. Las plataformas Coursera y FutureLearn ofertaron casi la mitad de los cursos sobre salud global. En cuanto al origen de los cursos, la mayoría fueron desarrollados por instituciones de Estados Unidos y del Reino Unido. Asimismo, casi la totalidad de cursos se dictan en idioma inglés. Sobre las opciones de subtítulos, menos de la mitad de los cursos presentaban traducciones a idiomas distintos del inglés. La mayoría de los cursos fueron de acceso gratuito. En torno al dominio de la salud global principalmente abordado por estos cursos, la globalización de la salud y la asistencia sanitaria fue el más desarrollado.

La oferta encontrada de cursos en línea señala que estos son una alternativa disponible para el aprendizaje sobre salud global, ya que son gratuitos y pueden dictarse en diferentes idiomas (subtítulos). Se resalta que el número de cursos sobre salud global encontrados es superior al descrito para otras áreas de la salud como la salud mental ^23^ y la educación en ciencias de la salud ^24^.

La oferta de cursos en línea permitiría a los interesados conocer sobre las distintas competencias requeridas en la formación sobre salud global, descritas por el CUGH ^18^.

De los cursos en línea en salud global encontrados en el presente estudio, existe un mayor número de cursos que cubren las competencias de globalización de la salud y la asistencia sanitaria; la creación de capacidades; la carga global de la enfermedad, y los determinantes sociales y ambientales de la salud. Aunque aún existen limitaciones en la oferta educativa en salud global, sobre todo en países de medianos y bajos ingresos, los cursos en línea serían una opción que tener en cuenta para el entrenamiento y formación continua de estudiantes y profesionales de la salud de estas regiones. Esto se ve reflejado en la contribución de los cursos en línea para el desarrollo profesional en países de Latinoamérica, donde su oferta y demanda sigue creciendo ^7^^,^^25^.

Específicamente en salud, las instituciones de países de altos ingresos, como Estados Unidos y Reino Unido, son las que lideran la elaboración de cursos en línea ^14^. Esto refleja la desigualdad en la enseñanza en áreas de la salud, además de la menor difusión y uso de tecnologías de la información y la comunicación, para el desarrollo de cursos en línea en países de bajos ingresos ^7^. Latinoamérica, el número de cursos en línea ofrecidos es bajo con respecto a otras regiones ^17^. Para el 2016, se reportó que menos del 10,6 % de universidades de Latinoamérica ofertan este tipo de cursos ^16^. Esto concuerda con la ausencia de cursos en línea sobre salud global elaborados por regiones de países de bajos y medianos ingresos encontrado en el presente estudio.

Respecto a la duración de los cursos, se encontró que estos, en promedio, demandaban unas cinco semanas, con una disponibilidad de cuatro horas y veinticuatro minutos por semana para culminar con las actividades de los cursos. Se ha reportado que los cursos ofertados por instituciones latinoamericanas cuentan con una duración promedio inferior a los ofertados en otras regiones ^16^. Asimismo, se conoce que las instituciones que los ofrecen los invierten más tiempo en la preparación del curso que el estimado para culminarlo ^11^.

Dado que los cursos en línea están pensados para abordar las competencias de un área en específica, la duración de estos cursos sería la adecuada para el estudiante de pregrado o profesional interesado en recibir entrenamiento en salud global. Por otro lado, la mayoría de los cursos no requieren que los participantes realicen un pago para acceder a los cursos, ya que están concebidos con la filosofía de democratizar el acceso a la información ^10^. En este sentido, los cursos en línea serían una herramienta útil de capacitación porque únicamente se requiere acceso a internet y un equipo que permita navegar por este servicio. Una persona interesada en capacitarse sobre salud global podría tomar un curso sin restricciones para su participación, tales como las barreras geográficas y económicas.

La mayoría de los cursos en línea en salud global se ofrecen en idioma inglés y son elaborados por reconocidas instituciones académicas de países anglosajones, como ha sido previamente indicado para otras áreas de salud ^11^^,^^14^. En el 2013, se reportó que el 94 % de los cursos en salud y medicina, se ofrecían en ese idioma ^14^. Si bien, se describe que la proporción de cursos en inglés se ha reducido ligeramente, pasando del 80 % en 2014 al 75 % en 2015 ^26^, este idioma sigue siendo el predominante en la oferta de cursos en línea, con un 82,5 % para el 2021 ^27^.

El aumento de proveedores de otras partes del mundo ha permitido que, hoy en día, se oferten cursos en línea en varios idiomas como español, francés, chino, árabe y portugués ^17^^,^^27^. Considerando que los cursos en línea buscan brindar un acceso a la información de forma global, se hace necesario que las instituciones que los ofertan los diversifiquen el idioma de los cursos, para que estos puedan ser utilizados por un número mayor de personas.

Como cualquier modelo de aprendizaje, los cursos en línea tienen sus problemas y sus desafíos. La formación de un grupo masivo de participantes es fundamental para encontrar un modelo de sostenibilidad de estas iniciativas, ya que hay un gran número de alumnos que no terminan los cursos ^27^. Además del alto costo de realizar un curso en línea, teniendo en cuenta que son gratuitos, son muchas las universidades que no apuestan por desarrollarlos porque no ven un retorno de la inversión. Sin embargo, los cursos en línea permiten una amplia visibilidad y posicionamiento tanto para los profesores como para las instituciones ^28^. Muchos estudiantes y educadores los reconocen como una experiencia de aprendizaje interesante, pero no necesariamente los reconocen como cursos con calidad, considerando que un curso debe estar más estructurado que un curso en línea ^11^. Asimismo, es importante que se promueva la creación de más cursos en línea en países de bajos y medianos ingresos como los de Latinoamérica, ofertando cursos con trascendencia en salud pública, en idiomas locales y, con una estructura y duración adecuada, ya que favorecen el desarrollo de mejores profesionales en estas regiones.

Entre las limitaciones de este estudio, se debe precisar que presenta un reporte histórico de los cursos en línea sobre salud global ofertados en distintas plataformas. Con ello, algunos de los cursos encontrados podrían no estar disponibles de forma ilimitada o en el momento actual, pues su oferta se da de acuerdo con las consideraciones de cada plataforma o institución. Por otro lado, los cursos en línea ofertados presentaron en sus contenidos más de un dominio sobre salud global, pero por criterio de los autores, sólo se consideró el principal. Sin embargo, consideramos que la búsqueda específica para cada una de las plataformas de los cursos en línea, así como en los sitios que los congregan de distintas plataformas, fue adecuado para identificar la oferta cursos en línea en salud global.

En conclusión, se encontró una importante oferta de cursos en línea sobre salud global. Estos cursos en su mayoría abordaron las competencias de globalización de la salud y asistencia sanitaria, fortalecimiento de capacidades, carga global de la enfermedad y determinantes sociales y ambientales de la salud. En general, la oferta de cursos en línea permitiría a estudiantes y profesionales de muchas regiones del mundo aprender sobre las distintas competencias requeridas de salud global. Sin embargo, sería adecuado aumentar la oferta de cursos en línea en diferentes idiomas, así como promover su desarrollo por parte de instituciones de países de bajos y medianos ingresos como los de Latinoamérica, los cuales suelen ser los más vulnerables frente a problemas de salud global y necesitan de tomadores de decisiones en salud pública que cuenten con la perspectiva desde la salud global.

## References

[1] Institute of Medicine (US) Committee on the US Commitment to Global Health (2009). The US Commitment to Global Health: Recommendations for the public and private sectors..

[2] Salm M, Ali M, Minihane M, Conrad P. (2021). Defining global health: findings from a systematic review and thematic analysis of the literature. BMJ Glob Health.

[3] Koplan JP, Bond TC, Merson MH, Reddy KS, Rodriguez MH, Sewankambo NK (2009). Towards a common definition of global health. Lancet.

[4] Orbinski J. (2004). Access to medicines and global health: will Canada lead or flounder?. CMAJ.

[5] Kovats RS. (2005). Global climate change and health: recent findings and future steps. CMAJ.

[6] Farmer PE, Furin JJ, Katz JT. (2004). Global health equity. Lancet.

[7] Solimano G, Valdivia L. (2014). Salud global en las instituciones académicas latinoamericanas: hacia un desarrollo e identidad propia. Saúde Soc.

[8] Bills CB, Ahn J. (2016). Global health and graduate medical education: a systematic review of the literature. J Grad Med Educ.

[9] Macfarlane SB, Jacobs M, Kaaya EE. (2008). In the name of global health: trends in academic institutions. J Public Health Policy.

[10] Hoy MB. (2014). MOOCs 101: An Introduction to Massive Open Online Courses. Med Ref Serv Q.

[11] Gooding I, Klaas B, Yager JD, Kanchanaraksa S. (2013). Massive open online courses in public health. Front Public Health.

[B12] Downes S. (2018). Places to go: connectivism & connective knowledge. Innov J Online Educ.

[13] Shah D. (2020). By The Numbers: MOOCs in 2020. The Report by ClassCentral.

[14] Liyanagunawardena TR, Williams SA. (2014). Massive open online courses on health and medicine: review. J Med Internet Res.

[15] Pérez-Álvarez R, Maldonado JJ, Rendich R, Pérez-Sanagustín MSD. (2017). Observatorio MOOC UC: la Adopción de MOOCs en la Educación Superior en América Latina y Europa.

[16] Manotas Salcedo E. (2019). Massive online courses, MOOC: courses for the whole minorities?: a review from positions about the impact of virtual education and the reducing of the social gap. Investigación y Desarrollo.

[17] Culquichicón C, Helguero-Santin LM, Labán-Seminario LM, Cardona-Ospina JA, Aboshady OA, Correa R. (2017). Massive open online courses in health sciences from Latin American institutions: A need for improvement?. F1000Research.

[18] Jogerst K, Callender B, Adams V, Evert J, Fields E, Hall T (2015). Identifying Interprofessional Global Health Competencies for 21st-Century Health Professionals. Ann Glob Health.

[19] Garcia PJ, Curioso WH, Lazo-Escalante M, Gilman RH, Gotuzzo E. (2009). Global health training is not only a developed-country duty. J Public Health Policy.

[20] Bendezu-Quispe G, Quijano-Escate R, Hernández-Vásquez A, Inga-Berrospi F, Cóndor DF. (2020). Massive Open Online Courses for continuing education for nursing professionals in Peru. Rev. Latino-Am. Enfermagem.

[21] Shah D. (2021). A Decade of MOOCs: A Review of MOOC Stats and Trends in 2021. The Report by Class Central.

[22] Bendezu-Quispe G, Quispe-Colquepisco S, Torres-Roman JS. (2016). Cursos masivos abiertos en línea y salud mental: una oportunidad de acercamiento a un problema desalud global. Rev Neurol.

[23] Longhini J, De Colle B, Rossettini G, Palese A. (2021). What knowledge is available on massive open online courses in nursing and academic healthcare sciences education? A rapid review. Nurse Educ Today.

[24] Banco Interamericano de Desarrollo Sigue en aumento el registro a MOOCs en América Latina y el Caribe.

[25] Hansen JD, Reich J. (2015). Democratizing education? Examining access and usage patterns in massive open online courses. Science.

[26] Shah D. By The Numbers: MOOCS in 2015 - Class Central.

[27] Capdevilla Pagès R, Aranzadi Elejabeitia P. (2014). Los cursos online masivos y abiertos: ¿oportunidad o amenaza para las universidades iberoamericanas?. RIED.

[28] Hollands FM, Tirthali D. (2014). MOOCs: expectations and reality. Full report. Center for BenefitCost Studies of Education.

